# Tofacitinib Suppresses Several JAK-STAT Pathways in Rheumatoid Arthritis *In Vivo* and Baseline Signaling Profile Associates With Treatment Response

**DOI:** 10.3389/fimmu.2021.738481

**Published:** 2021-09-24

**Authors:** Maaria Palmroth, Krista Kuuliala, Ritva Peltomaa, Anniina Virtanen, Antti Kuuliala, Antti Kurttila, Anna Kinnunen, Marjatta Leirisalo-Repo, Olli Silvennoinen, Pia Isomäki

**Affiliations:** ^1^ Molecular Immunology Group, Faculty of Medicine and Health Technology, Tampere University, Tampere, Finland; ^2^ Department of Bacteriology and Immunology, Faculty of Medicine, University of Helsinki, Helsinki, Finland; ^3^ Department of Rheumatology, University of Helsinki and Helsinki University Hospital, Helsinki, Finland; ^4^ Centre for Rheumatic Diseases, Tampere University Hospital, Tampere, Finland; ^5^ Fimlab Laboratories, Pirkanmaa Hospital District, Tampere, Tampere, Finland; ^6^ Institute of Biotechnology, HiLIFE Helsinki Institute of Life Sciences, University of Helsinki, Helsinki, Finland

**Keywords:** rheumatoid arthritis, cytokines, JAK inhibitor, monocytes, T cells, B cells

## Abstract

**Objective:**

Current knowledge on the actions of tofacitinib on cytokine signaling pathways in rheumatoid arthritis (RA) is based on *in vitro* studies. Our study is the first to examine the effects of tofacitinib treatment on Janus kinase (JAK) - signal transducer and activator of transcription (STAT) pathways *in vivo* in patients with RA.

**Methods:**

Sixteen patients with active RA, despite treatment with conventional synthetic disease-modifying antirheumatic drugs (csDMARDs), received tofacitinib 5 mg twice daily for three months. Levels of constitutive and cytokine-induced phosphorylated STATs in peripheral blood monocytes, T cells and B cells were measured by flow cytometry at baseline and three-month visits. mRNA expression of JAKs, STATs and suppressors of cytokine signaling (SOCS) were measured from peripheral blood mononuclear cells (PBMCs) by quantitative PCR. Association of baseline signaling profile with treatment response was also investigated.

**Results:**

Tofacitinib, in csDMARDs background, decreased median disease activity score (DAS28) from 4.4 to 2.6 (p < 0.001). Tofacitinib treatment significantly decreased cytokine-induced phosphorylation of all JAK-STAT pathways studied. However, the magnitude of the inhibitory effect depended on the cytokine and cell type studied, varying from 10% to 73% inhibition following 3-month treatment with tofacitinib. In general, strongest inhibition by tofacitinib was observed with STAT phosphorylations induced by cytokines signaling through the common-γ-chain cytokine receptor in T cells, while lowest inhibition was demonstrated for IL-10 -induced STAT3 phosphorylation in monocytes. Constitutive STAT1, STAT3, STAT4 and STAT5 phosphorylation in monocytes and/or T cells was also downregulated by tofacitinib. Tofacitinib treatment downregulated the expression of several JAK-STAT pathway components in PBMCs, SOCSs showing the strongest downregulation. Baseline STAT phosphorylation levels in T cells and monocytes and SOCS3 expression in PBMCs correlated with treatment response.

**Conclusions:**

Tofacitinib suppresses multiple JAK-STAT pathways in cytokine and cell population specific manner in RA patients *in vivo*. Besides directly inhibiting JAK activation, tofacitinib downregulates the expression of JAK-STAT pathway components. This may modulate the effects of tofacitinib on JAK-STAT pathway activation *in vivo* and explain some of the differential findings between the current study and previous *in vitro* studies. Finally, baseline immunological markers associate with the treatment response to tofacitinib.

## Introduction

Cytokines are important mediators of inflammation and tissue destruction in rheumatoid arthritis (RA) (1, 2). Several cytokines involved in the pathogenesis of RA, such as interleukin-6 (IL-6), interferons (IFNs), granulocyte-macrophage colony-stimulating factor (GM-CSF) and common gamma chain cytokine family, act through Janus kinase (JAK)-signal transducer and activator of transcription (STAT) pathway (3, 4). JAK-STAT pathways consist of four JAK kinases [JAK1-3 and tyrosine kinase 2 (TYK2)] and seven signal transducers and activators of transcription (STAT1-6, including the homologs STAT5A and STAT5B). The signaling cascade is initiated by a cytokine binding to its receptor, which enables JAK activation by trans-phosphorylation. Subsequently, JAKs phosphorylate the receptor and STATs that dimerize, and translocate to the nucleus to regulate the expression of their target genes. Each cytokine receptor employs a specific combination of JAK kinases, e.g. IL-6 signals through JAK1 and JAK2/TYK2 and common gamma chain cytokines through JAK1 and JAK3 (5). JAK-STAT signaling pathway is under tight regulation, which involves e.g. proteins of suppressors of cytokine signaling (SOCS) family SOCS1-3 and cytokine-inducible SH2-containing protein (CIS1) (6).

We and others have demonstrated that certain JAK-STAT pathways are constitutively active in rheumatic diseases (7–10). In RA, STAT3 is constitutively phosphorylated in circulating T cells and monocytes, and this correlates with serum IL-6 levels, suggesting hyperactivation of the IL-6 –STAT3 axis (7, 9). In addition to STAT3, constitutive phosphorylation of STAT1 and STAT5 is increased in peripheral blood T cells from patients with active RA (7, 10). Constitutive STAT3 phosphorylation in circulating T cells of patients with recent-onset RA associates with disease activity and good treatment response to conventional synthetic disease-modifying antirheumatic drugs (csDMARDs) (8). In addition, we have demonstrated that cytokine-induced STAT1 and STAT6 phosphorylation in circulating leukocytes associates with treatment response to biological drugs in established RA and to csDMARDs in recent-onset RA, respectively (11).

Tofacitinib, which inhibits JAK1, JAK3, and to a slightly lesser extent JAK2, was the first JAK inhibitor developed for treatment of RA (12, 13) and its efficacy is comparable to that of TNF-inhibitor adalimumab (14). Tofacitinib has been shown to inhibit cytokine signaling and the effector functions of different immune cells and synovial fibroblasts *in vitro* (15–26) and in animal models of arthritis (15, 27, 28). However, information of the *in vivo* effects of tofacitinib on the activity of JAK-STAT pathways in RA patients is currently lacking and may differ from the results based on *in vitro* studies.

Gaining knowledge on how medicines, such as tofacitinib, function in patients with RA could help us to understand disease mechanisms, as well as both desired and undesired effects of JAK inhibitors. The current study is the first to investigate the JAK-STAT signaling profile by flow cytometry, and the expression of the signaling pathway components, in peripheral blood leukocytes of tofacitinib-treated RA patients. In addition, we examined associations between baseline immunological findings and the treatment response to tofacitinib.

## Materials and Methods

### Patients

Patients fulfilling the 2010 ACR/EULAR classification criteria for RA were recruited for this clinical study in two rheumatology outpatient clinics (Tampere and Helsinki University Hospitals) between June 2018 and January 2020. Eligible patients had active disease at baseline visit: Disease Activity Score for 28 joints based on the C-reactive protein level (DAS28-4[CRP]) was >3.2 despite treatment with methotrexate and/or other csDMARDs. Key exclusion criteria were prior treatment with biologic therapies or JAK inhibitors, current infection, malignancy, severe hepatic impairment, pregnancy or lactation, hemoglobin <90 mg/dl, neutrophil count <1.0 × 109/l or lymphocyte count <0.75 x 109/l.

This study was approved by the National Committee on Medical Research Ethics (TUKIJA) and Finnish Medicines Agency Fimea and was conducted according to the principles of the Declaration of Helsinki and Good Clinical Practice Guidelines. All patients gave their written informed consent.

### Study Design

**Figure 1:**
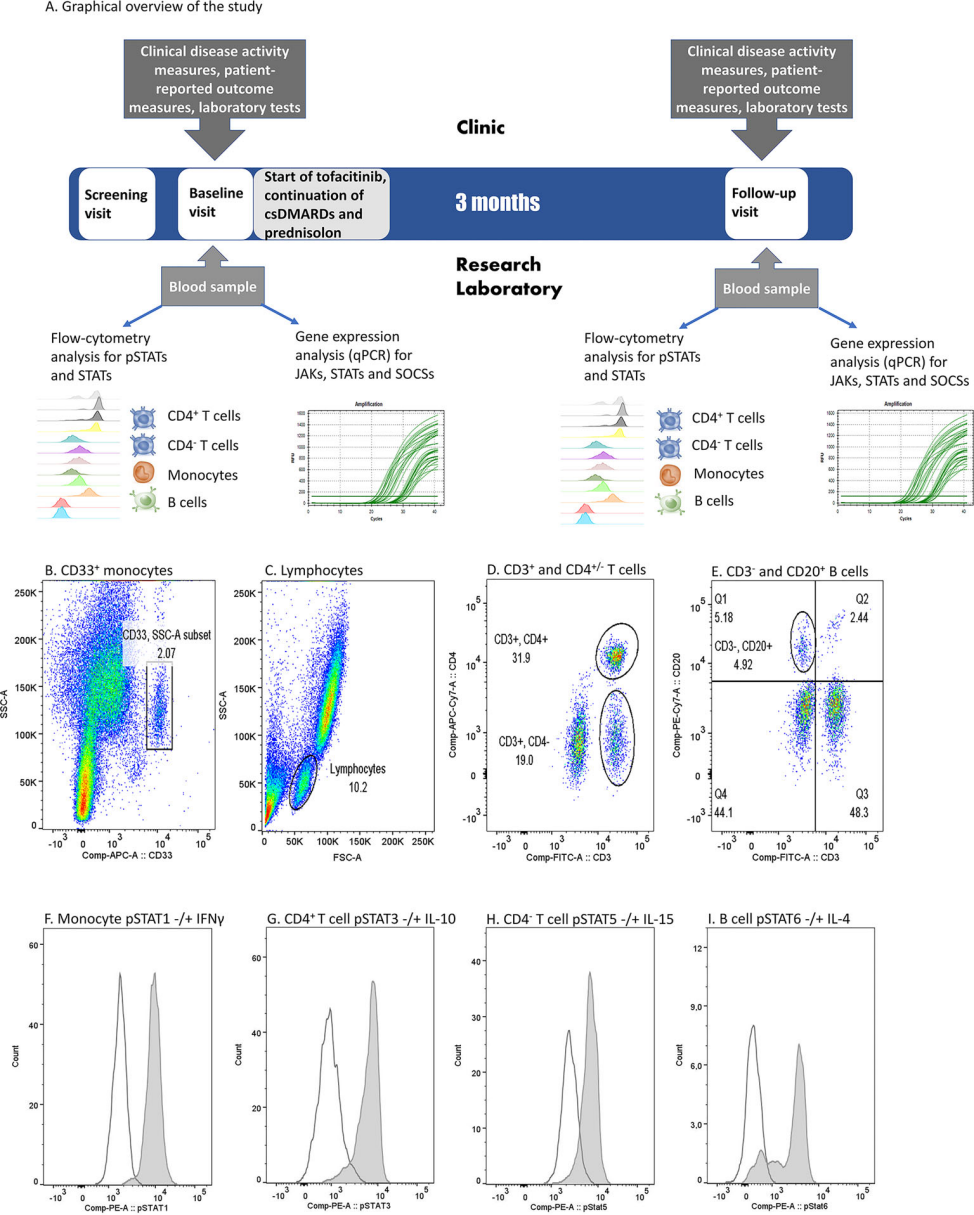
**(A)** Graphical overview of the study and **(B–I)**. Flow cytometry gating strategy and examples of cytokine-induced phosphorylated STAT (pSTAT) in each cell population in one patient sample. **(B)** Monocyte gate was set based on CD33 positivity and light scattering characteristics (SSC-A). **(C)** Lymphocytes were gated based on light scattering characteristics (FSC-A and SSC-A). **(D)** Among lymphocytes, T cell gates were set to comprise CD3^+^CD4^+^ or CD3^+^CD4^-^ populations. **(E)** Among lymphocytes, B cell gate was set to comprise CD3^-^ and CD20^+^ population. **(F)** pSTAT1 histograms in unstimulated (open peak) and IFN-γ-stimulated (grey peak) monocytes. **(G)** pSTAT3 histograms in unstimulated (open peak) and IL-10-stimulated (grey peak) CD4^+^ T cells. **(H)** pSTAT5 histograms in unstimulated (open peak) and IL-15-stimulated (grey peak) CD4^-^ T cells. **(I)** pSTAT6 histograms in unstimulated (open peak) and IL-4 stimulated (grey peak) B cells. IFN, interferon; IL, interleukin, (p)STAT, (phosphorylated) signal transducer and activator of transcription.

Graphical overview of the study is presented in **Figure 1A**. The study included a screening visit (0-3 months before baseline visit), a baseline visit (0 month) and a follow-up visit (3 months). Screening visit and baseline visit could be combined. Tofacitinib 5 mg twice daily was started at baseline visit and continued through the study. Patients continued their csDMARD therapy and prednisolone (0-10 mg/day) at a stable dose during the study.

The following patient-reported outcomes and clinical assessments were recorded at study visits: patient general health visual analogue scale (VAS) (0-100 mm), pain VAS (0-100 mm), health assessment questionnaire (HAQ) disability index (0-3), number of swollen and tender joints (46 joint count), physician’s assessment VAS (0-100 mm) and DAS28. Tofacitinib treatment response was determined by the change from baseline DAS28-4[CRP].

Study-related blood samples were drawn at baseline and follow-up visits. Blood samples at the follow-up visit were taken 1-2 hours after the morning tofacitinib dose.

### Cytokine-Stimulations and Flow Cytometry

Phosphorylation of STAT proteins, and levels of total STAT1 and STAT3 were studied using five-color flow cytometry. First, 100-µl aliquots of fresh blood samples were either left unstimulated or were stimulated by 100 ng/ml recombinant IL-2, IL-4, IL-6, IL-7, IL-10, IL-15, IL-21, IFN-α or IFN-γ (listed in detail in **Supplementary Table 1**) for 15 minutes at +37°C. The stimulation was terminated by transferring the samples on ice. Leukocytes were then immediately fixed and red blood cells lysed using BD Phosflow Lyse/Fix buffer (Becton, Dickinson and Company, Franklin Lakes, NJ, USA) for 10 min at +37°C, washed with PBS and permeabilized in ice-cold methanol for 10 min on ice followed by 1-4 weeks preservation in methanol at -80°C. In order to ensure the integrity of the results the samples that were collected in Helsinki were transferred in methanol on dry ice to Tampere, where sample preparation was continued and all stainings were performed. STAT phosphorylation levels were determined to be preserved for at least 4 weeks in methanol at -80°C in preliminary experiments (unpublished data).

Following two washes with FACS buffer (PBS supplemented with 0,1% bovine serum albumin and 0,01% sodium azide), samples were stained with fluorochrome-conjugated antibodies against CD33, CD3, CD4, CD20, STAT1, STAT3, phospho-STAT1 (pSTAT1), pSTAT3, pSTAT4, pSTAT5 and pSTAT6 (listed in detail in **Supplementary Table 2**) for 30 minutes at room temperature, protected from light. CD33 marker was selected to represent monocytes and CD20 to represent B cells, as the epitopes of more conventionally used markers CD14 and CD19, respectively, do not survive cold methanol permeabilization (29). Following antibody stainings the samples were washed twice with FACS buffer and fluorescence was measured with FACS Canto II (BD). To ensure consistent performance of the method throughout the study, 8-peak Rainbow calibration particles (BD) were used before every run.

Data acquisition was performed using FACSCanto II (BD) and the analysis of flow cytometer data using FlowJo Single cell analysis software (BD). CD4^+^ and CD4^-^ T cells were gated from the CD3^+^ lymphocyte population, CD20^+^ B cells from the CD3^-^ lymphocyte population and CD33^+^ cells represent monocytes (**Figures 1B–E**). A phycoerythrin (PE) fluorescence histogram was created for each cell population, and the median fluorescence intensity (MFI, arithmetic median) was calculated. Examples of cytokine-induced pSTATs are presented in **Figures 1F–I**. The change in STAT phosphorylation during the study was calculated by dividing the difference of fluorescence intensities at entry and after 3 months and dividing that by the fluorescence intensity at entry.

### Peripheral Blood Mononuclear Cell Isolation, RNA Extraction, and qPCR Analysis

Peripheral blood mononuclear cells (PBMCs) were isolated by density gradient centrifugation using Histopaque 1077 medium (Sigma Aldrich, St. Louis, MO, USA), washed twice with PBS containing 2 mM EDTA and snap frozen. Samples were collected at both locations but transferred from Helsinki to Tampere on dry ice for further preparations and analysis. Total RNA was extracted from PBMCs using the RNeasy Mini-Kit (Qiagen, Valencia, CA, USA). Total RNA (0.5 µg) was reverse-transcribed using M-MLV reverse transcriptase (Thermo Scientific, Waltham, MA, USA) according to the manufacturer’s instructions. Quantitative PCR (qPCR) reactions were performed by using HOT FIREPol EvaGreen qPCR Mix Plus (Solis BioDyne, Tartu, Estonia). Primer sequences for *STAT1, STAT3, STAT4, STAT5A, STAT5B, STAT6, JAK1, JAK2, JAK3, TYK2, SOCS1, SOCS2, SOCS3, CIS1* and *β-actin* are listed in **Supplementary Table 3**. The 10-µl real-time PCR reactions were performed with CFX384 (Bio-Rad Laboratories, Hercules, CA, USA) and gene expression was quantified by using the delta C(T) method by normalizing to the expression of *β-actin*.

### Statistical Methods

Continuous variables are summarized as means with ranges or bootstrapped 95% confidence intervals (CI), or medians with interquartile ranges (IQR). Values measured at study entry and after 3 months were compared using Wilcoxon signed rank test. Correlation between variables was assessed by Spearman rank correlation. No adjustment was made for multiple testing and p-values equal or less than 0.05 were considered statistically significant. Statistical analysis was carried out using SPSS version 25 (IBM, Armonk, NY, USA) and Stata version 15 (StataCorp LLC, College Station, TX, USA).

## Results

### Patients

Eighteen patients, 9 from each outpatient clinic, were recruited to the study. Of these, one patient did not start tofacitinib treatment and for another patient flow cytometry results could not be obtained at baseline visit due to a technical problem with the flow cytometer. The final study population therefore consisted of 16 patients, who continued tofacitinib treatment until follow-up visit and had complete data sets from both visits.

Characteristics of the patient cohort at baseline visit are presented in **Table 1**. The background csDMARD therapy of each patient is described in detail in **Supplementary Table 4**. A total of 12 patients (75%) received methotrexate as part of their csDMARD regimen (6 with triple, 4 with double, and 2 with single csDMARD therapy).

**Table 1:** Characteristics of the patients (n=16).

Female sex, n (%)	11 (69%)
Age, years, mean (range)	58.4 (36.6-72.9)
Disease duration, years, mean (range)	9.6 (0.5-48.0)
Rheumatoid factor positive, n (%)	11 (69%)
CCP-antibody positive, n (%)	12 (75%)
Erosive disease, n (%)	7 (44%)
Disease activity (DAS28), n (%)	
Moderate	13 (81%)
High	3 (19%)
csDMARD regimen, n (%)	
Triple	6 (37%)
Double	7 (44%)
Single	3 (19%)
Low-dose prednisolone, n (%)	8 (50%)

CCP, cyclic citrullinated peptide; DAS28, composite Disease Activity Score for 28 joints based on the C-reactive protein level (DAS28-4[CRP]); csDMARD, conventional systemic disease-modifying antirheumatic drug; n, number of patients.

### Clinical Response to Tofacitinib

Tofacitinib with background csDMARD treatment significantly decreased the activity of RA during 3-month treatment, as defined both by clinical measures of activity, as well as by patient-reported outcomes (**Table 2**). Median DAS28 score decreased from 4.4 to 2.6. Nine patients (56%) were in DAS28 remission at the 3-month visit. Four patients had low, two patients moderate and one patient high disease activity according to DAS28 following the treatment with tofacitinib and csDMARDs.

**Table 2:** Comparison of clinical and laboratory parameters at baseline and after 3-month treatment with tofacitinib and csDMARDs.

	Before, median (IQR)	After 3 months, median (IQR)	p
Swollen joint count, 0-46	7 (6-9)	2 (0-3)	**<0.001**
Tender joint count, 0-46	11 (5-17)	1 (0-8)	**<0.001**
Swollen joint count, 0-28	5 (4-7)	1 (0-2)	**<0.001**
Tender joint count, 0-28	4 (2-10)	1 (0-3)	**<0.001**
General health, VAS, 0-100 mm	51 (37-65)	16 (7-29)	**0.001**
Pain, VAS, 0-100 mm	43 (22-64)	12 (5-38)	**0.002**
Physician’s assessment, VAS, 0-100 mm	35 (31-46)	13 (11-18)	**<0.001**
HAQ disability index, 0-3	0.813 (0.625-1.253)	0.130 (0-0.813)	**0.011**
DAS28	4.4 (3.6-4.9)	2.6 (1.9-2.9)	**<0.001**
Plasma C-reactive protein, mg/l	5 (3-17)	3 (3-4)	**0.042**
Blood hemoglobin, g/l	129 (126-140)	132 (126-145)	0.775
Blood leukocyte count, ×10^9^/l	8.3 (6.1-9.1)	5.3 (4.3-6.9)	**0.003**
Blood neutrophil count, ×10^9^/l	5.32 (4.02-6.34)	2.88 (2.32-3.91)	**0.003**
Blood lymphocyte count, ×10^9^/l	1.40 (1.07-1.94)	1.41 (1.20-1.83)	0.959
Blood platelet count, ×10^9^/l	297 (274-334)	281 (225-302)	**<0.001**
Plasma alanine aminotransferase, U/l	20 (15-26)	21 (19-28)	0.224
Plasma creatinine, µmol/l	63 (56-83)	68 (56-86)	0.615

p-values are calculated using Wilcoxon test and shown in bold when p ≤ 0.05.

DAS28, composite Disease Activity Score for 28 joints based on the C-reactive protein level (DAS28-4[CRP]); csDMARD, conventional systemic disease-modifying antirheumatic drug; HAQ, Health Assessment Questionnaire; IQR, interquartile range; VAS, visual analogue scale.

Safety laboratory tests remained within acceptable range (**Table 2**) and there were no serious adverse events during the study.

### Tofacitinib Suppresses Both Constitutive and Cytokine-Induced STAT Phosphorylation *In Vivo* in Cytokine and Cell Type Specific Matter

**Figure 2:**
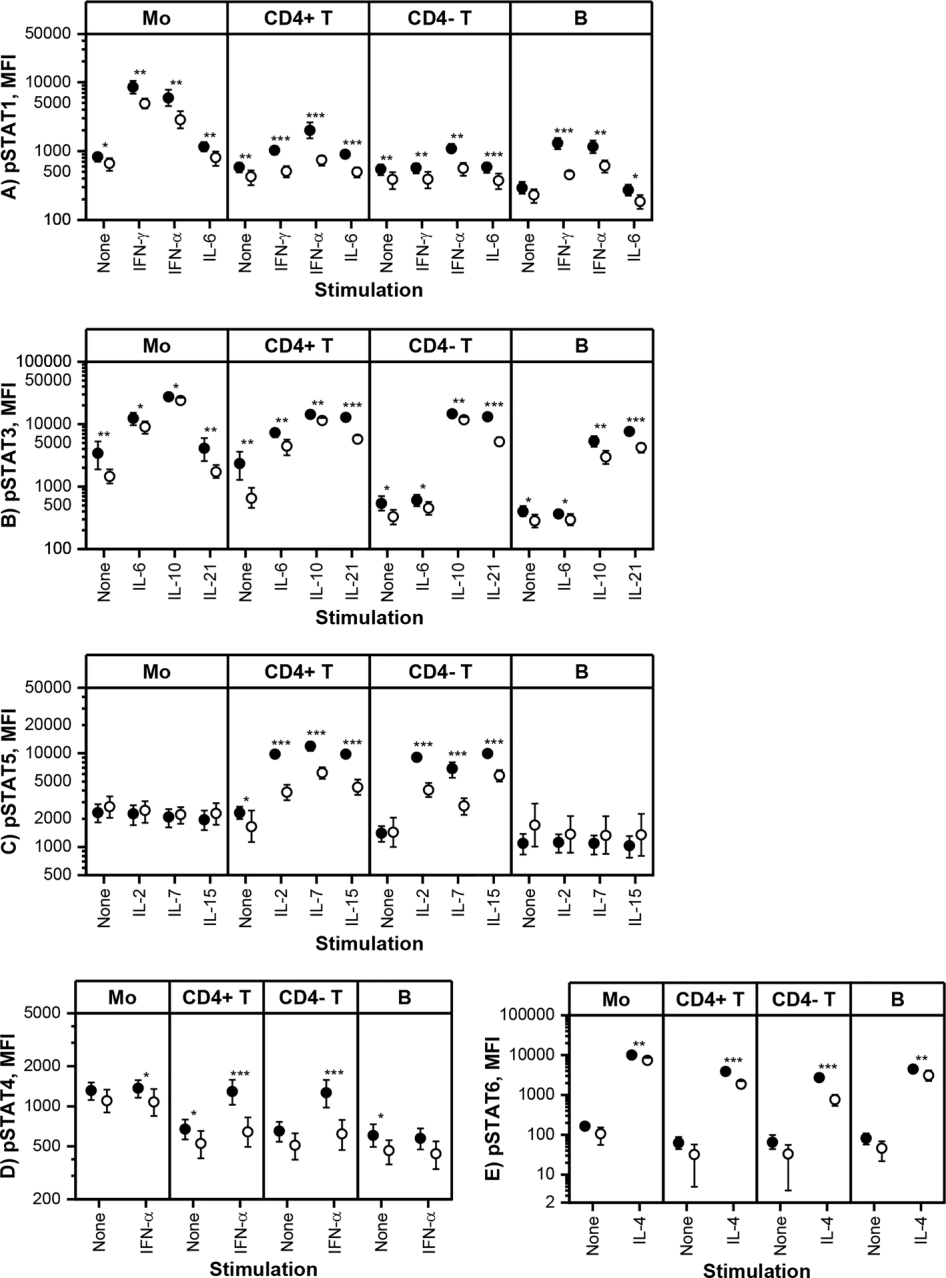
Means and 95% confidence intervals of **(A)** phosphorylated STAT1 (pSTAT1), **(B)** pSTAT3, **(C)** pSTAT5, **(D)** pSTAT4 and **(E)** pSTAT6 median fluorescence intensities (MFI) in monocytes (Mo), CD4^+^ T cells (CD4^+^ T), CD4^-^ T cells (CD4^-^ T) and CD20^+^ B cells (B) at baseline (filled symbols) and after 3-month treatment with tofacitinib and csDMARDs (open symbols). Note the logarithmic scale of the Y-axis. p-values comparing 0 and 3 months are calculated by Wilcoxon test. Significant differences are marked with an asterisk: *p ≤ 0.05, ** p ≤ 0.01, p***≤ 0.001. csDMARD, conventional systemic disease-modifying antirheumatic drug; (p)STAT, (phosphorylated) signal transducer and activator of transcription.

Constitutive and *in vitro* cytokine-induced STAT phosphorylation in monocytes, T cells and B cells was measured using multi-color flow cytometry at baseline and three-month visits. Based on the pharmacokinetic profile of tofacitinib (29), our results describe the maximal *in vivo* effect of tofacitinib, as the blood samples at three-month visit were collected shortly after morning tofacitinib dose. The flow cytometric assay was performed using fresh blood without cell isolation, and thus is likely to reflect STAT phosphorylation *in vivo*. Cytokine-induced STAT phosphorylation was studied using selected cytokine or cytokines for each pSTAT (**Figure 2**).

Tofacitinib, in csDMARD background, decreased significantly constitutive phosphorylation of STATs (**Figure 2**). The decrease in constitutive phosphorylation was most consistent for STAT3 (shown in all cell types studied). In addition, constitutive phosphorylation of STAT1, STAT4 and STAT5 was also downregulated by tofacitinib in a cell type specific manner. Using similar methodology as in the current study, we have previously shown that constitutive pSTAT1, pSTAT3 and pSTAT5 are increased in circulating T cells and pSTAT3 in monocytes of RA patients compared to healthy volunteers (7, 10). In order to gain some insight into what extent these leukocyte signaling aberrations are normalized by tofacitinib treatment, we compared the differences in constitutive STAT phosphorylation between controls and RA patients (historical data) to those between tofacitinib-treated and untreated RA patients derived from the current study (**Supplementary Table 5**). This comparison suggests that the elevated pSTAT1, pSTAT3 and pSTAT5 levels in patients with RA are reversed to a significant degree by treatment with tofacitinib.

Cytokine-induced STAT phosphorylations were significantly decreased by tofacitinib in all studied leukocyte subtypes (**Figure 2**). In order to investigate the inhibitory potency of tofacitinib on STAT activation induced by different cytokines more closely, mean inhibition percentages between 3-month and baseline visits were calculated (**Table 3**). Depending on the cytokine and cell population, the mean percentage inhibition of STAT phosphorylation by tofacitinib treatment ranged from 10% to 73%. At least 50% inhibition was observed with STAT phosphorylations induced by IFN-α and common-γ-chain cytokines IL-2, IL-4, IL-15 and IL-21 in CD4^+^ T cells, by IL-2, IL-4 and IL-21 in CD4^-^ T cells and by IFN-γ in B cells. Lowest inhibition was demonstrated for IL-10 -induced STAT3 phosphorylation in monocytes. In general, monocyte responses were less sensitive to suppression by tofacitinib than those in CD4^+^ T cells.

**Table 3:** Mean (95% confidence interval) percentage inhibition of cytokine-induced STAT phosphorylation by tofacitinib in different cell populations at three months compared to baseline.

Cytokine	JAKs	STAT	Percentage inhibition in different cell types			
			Monocytes	CD4^+^ T cells	CD4^-^ T cells	B cells
IFN-γ	JAK1/2	pSTAT1	34 (22;45)	49 (40;58)		**58** (45;68)
IFN-α	JAK1/TYK2	pSTAT1	34 (7;54)	**50** (35;63)	43 (31;56)	30 (0.4;52)
IFN-α	JAK1/TYK2	pSTAT4		45 (34;56)	45 (34;56)	
IL-2	JAK1/3	pSTAT5		**60** (50;69)	**54** (44;64)	
IL-4	JAK1/3	pSTAT6	25 (14;36)	**53** (44;61)	**73** (65;80)	25 (3;52)
IL-7	JAK1/3	pSTAT5		47 (37;57)	47 (25;63)	
IL-15	JAK1/3	pSTAT5		**55** (43;62)	40 (29;50)	
IL-21	JAK1/3	pSTAT3		**55** (47;62)	**60** (53;67)	42 (33;53)
IL-6	JAK1/2/TYK2	pSTAT1	29 (18;39)	41 (31;51)		
IL-6	JAK1/2/TYK2	pSTAT3	12 (-30;45)	42 (27;57)	22 (9;35)	
IL-10	JAK1/TYK2	pSTAT3	10 (-1;21)	19 (12;26)	19(10;27)	43 (28;56)

Results with percentages ≥ 50 are shown in bold. Spaces left empty denote the cases in which the stimulated phosphorylation level does not differ from the constitutive level. JAK proteins involved in each cytokine signaling pathway studied are presented in the Table.

IFN, interferon; IL, interleukin; JAK, Janus kinase; (p)STAT, (phosphorylated) signal transducer and activator of transcription; TYK2, tyrosine kinase 2.

### Tofacitinib Downregulates the mRNA Expression of JAK-STAT Signaling Pathway Components

**Figure 3:**
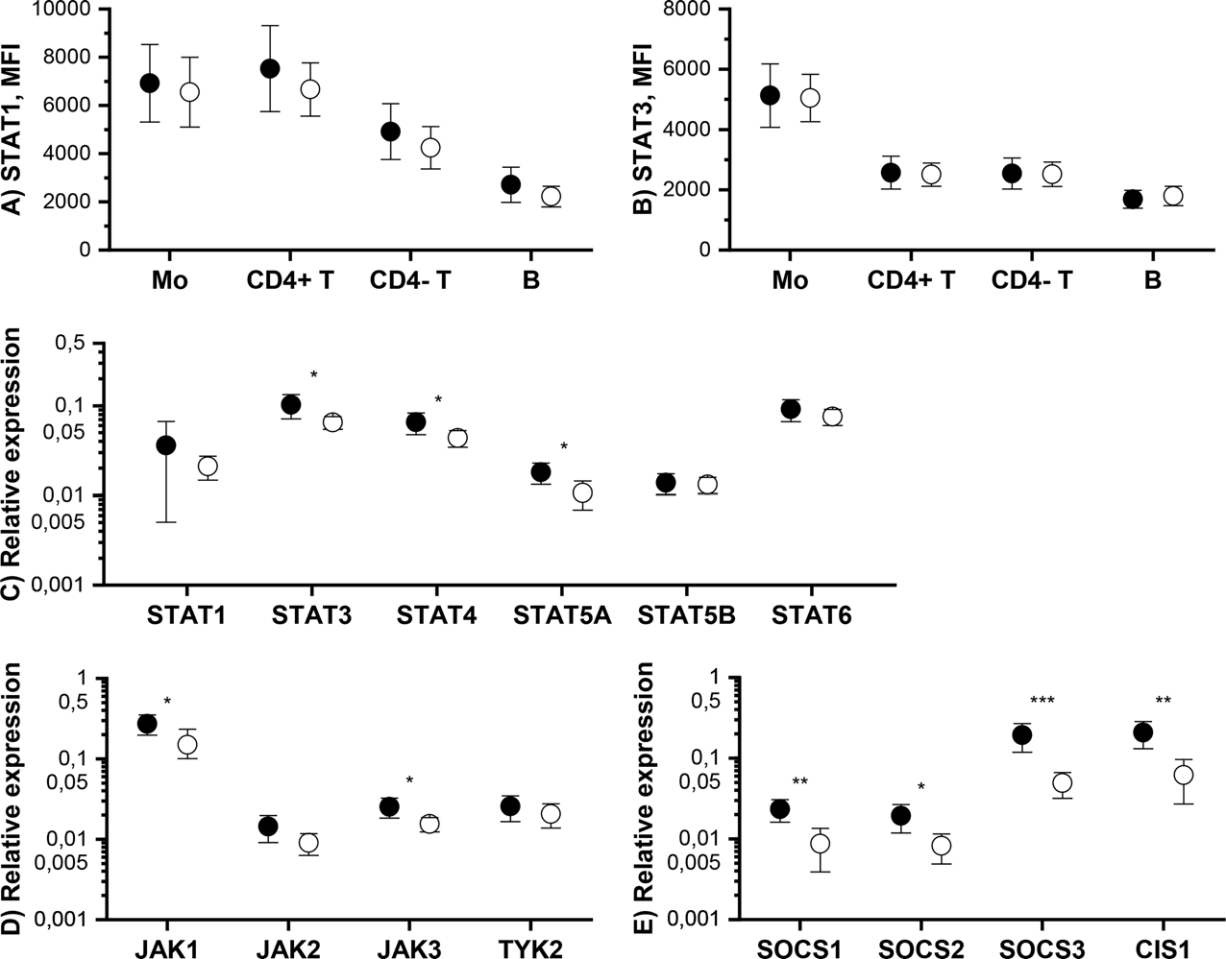
Means and 95% confidence intervals of total **(A)** STAT1 and **(B)** STAT3 protein expression level in monocytes (Mo), CD4^+^ T cells (CD4^+^ T), CD4^-^ T cells (CD4^-^ T) and B cells and mRNA expression levels of **(C)** STAT, **(D)** JAK and **(E)** SOCS genes in PBMCs at baseline (filled symbols) and after 3-month treatment with tofacitinib and csDMARDs (open symbols). p-values comparing 0 and 3 months are calculated by Wilcoxon test. Significant differences are marked with an asterisk: *p ≤ 0.05, ** p ≤ 0.01, p***≤ 0.001. CIS1, cytokine-inducible SH2 domain-containing protein; csDMARD, conventional systemic disease-modifying antirheumatic drug; PBMC, peripheral blood mononuclear cell; SOCS, suppressor of cytokine signaling; STAT, signal transducer and activator of transcription.

We also measured the total amount of STAT1 and STAT3 proteins in monocytes, T cells and B cells by flow cytometry. Tofacitinib did not cause significant changes in total STAT1 and STAT3 protein levels (**Figures 3A, B**).

The effect of tofacitinib on the mRNA levels of JAK-STAT pathway components and inhibitors in PBMCs was studied using qPCR. Significant, but rather modest, decreases were observed in the expression of *STAT3*, *STAT4*, *STAT5A*, *JAK1*, *JAK3* by tofacitinib, whereas there was no effect on *STAT1*, *STAT5B*, *STAT6, JAK2* and *TYK2* expression (**Figures 3C, D**). In contrast, the expression of all *SOCS*s studied was significantly downregulated during treatment with tofacitinib and csDMARDS (**Figure 3E**).

### Baseline STAT Phosphorylation in Monocytes and T Cells and Expression of SOCS3 in PBMCs Correlate With Treatment Response

To study whether baseline signaling profile is associated with treatment response to tofacitinib, correlation coefficients were calculated between baseline pSTATs, total STAT1 and STAT3 and the change from baseline in DAS28 after 3 months of treatment with tofacitinib and csDMARDs (**Table 4**).

**Table 4:** Correlation of baseline pSTAT levels and total STAT1 and STAT3 expression levels with improvement in DAS28 during 3-month treatment with tofacitinib and csDMARDs.

Molecule	Stim.	Cell type							
		Monocytes		CD4^+^ T cells		CD4^-^ T cells		B cells	
		r	p	r	p	r	p	r	p
pSTAT1	None	**0.591**	**0.016**	**0.603**	**0.013**	0.391	0.134	0.280	0.294
pSTAT1	IFN-γ	0.379	0.147	**0.532**	**0.034**			0.062	0.820
pSTAT1	IFN-α	-0.079	0.770	-0.150	0.579	-0.088	0.745	0.038	0.888
pSTAT1	IL-6	**0.556**	**0.025**	0.282	0.289				
pSTAT3	None	**0.635**	**0.008**	**0.732**	**0.001**	0.424	0.102	0.426	0.099
pSTAT3	IL-6	0.385	0.141	**0.621**	**0.010**	0.126	0.641		
pSTAT3	IL-10	0.253	0.345	0.224	0.405	0.185	0.492	0.224	0.405
pSTAT3	IL-21			0.115	0.672	-0.009	0.974	0.085	0.753
pSTAT4	None	**-0.506**	**0.046**	-0.491	0.053	**-0.518**	**0.040**	-0.344	0.192
pSTAT4	IFN-α			**-0.568**	**0.022**	**-0.638**	**0.008**		
pSTAT5	None	0.350	0.184	**0.568**	**0.022**	0.271	0.311	0.276	0.300
pSTAT5	IL-2			0.147	0.587	0.159	0.557		
pSTAT5	IL-7			0.026	0.922	-0.147	0.587		
pSTAT5	IL-15			0.162	0.549	0.147	0.587		
pSTAT6	None	-0.175	0.517	-0.130	0.633	-0.219	0.414	0.218	0.418
pSTAT6	IL-4	-0.082	0.762	-0.300	0.259	-0.412	0.113	-0.279	0.295
STAT1	None	-0.291	0.274	-0.356	0.176	-0.488	0.055	-0.212	0.431
STAT3	None	-0.307	0.265	-0.354	0.196	-0.479	0.071	-0.432	0.108

Spearman correlation coefficients (r) are used. Results with p-values ≤0.05 are shown in bold. Spaces left empty denote the cases in which the stimulated phosphorylation level does not differ from the constitutive level.

DAS28, composite Disease Activity Score for 28 joints based on the C-reactive protein level (DAS28-4[CRP]); csDMARD, conventional systemic disease-modifying antirheumatic drug; IFN, interferon; IL, interleukin; (p)STAT, (phosphorylated) signal transducer and activator of transcription; stim., stimulation.

Constitutive pSTAT1 and pSTAT3 in monocytes and constitutive pSTAT1, pSTAT3 and pSTAT5 in CD4^+^ T cells correlated positively with treatment response (**Supplementary Figures 1A–D, G**), while constitutive STAT4 phosphorylation in monocytes or in CD4^-^ T cells correlated negatively with treatment response (**Supplementary Figures 1E, F**). In addition, IL-6-stimulated pSTAT1 levels in monocytes, IFN-γ-stimulated pSTAT1 levels in CD4^+^ T cells and IL-6-stimulated pSTAT3 in CD4^+^ T cells correlated positively with treatment response. IFN-α-stimulated pSTAT4 in T cells correlated negatively with treatment response.

In addition, we studied the correlations between baseline demographic, clinical or laboratory variables and response to treatment with tofacitinib and csDMARDs. DAS28 (r=0.549; p=0.028) and CRP level (r=0.554; p=0.026) significantly associated with treatment response while the other parameters showed no correlation (**Supplementary Table 6**).

The association between the decrease in pSTAT or STAT levels during treatment and tofacitinib treatment response was also investigated. Decrease in constitutive STAT3 phosphorylation in monocytes (r =0.600, p=0.014) and in CD4^+^ T cells (r=0.682, p=0.004) correlated positively with treatment response (**Supplementary Table 7**).

Finally, correlation coefficients were calculated between baseline *STAT*, *JAK* and *SOCS* mRNA levels in PBMC, or the change in mRNA expression levels, and the tofacitinib treatment response (**Supplementary Table 8**). Baseline *SOCS3* levels correlated positively with treatment response (r = 0.532; p = 0.034).

## Discussion

In this study we show that in patients with chronic csDMARD-unresponsive rheumatoid arthritis, tofacitinib suppresses multiple JAK-STAT pathways *in vivo* by decreasing both constitutive and cytokine-induced STAT phosphorylation in circulating leukocytes. However, the level of suppression by tofacitinib depends on the cytokine and cell type studied and is somewhat different from that reported in previous *in vitro* studies. Moreover, tofacitinib inhibits mRNA expression of several JAK-STAT pathway components and inhibitory proteins. We also show that baseline pSTAT levels in monocytes and T cells and *SOCS3* level in PBMCs correlate with treatment response.

The selectivity of JAK inhibitors is currently a major subject of interest. Recently, three *in vitro* articles comparing the potencies of several JAK inhibitors towards different cytokine signaling pathways in PBMC or blood of healthy donors have been published (17, 18, 26). Even though *in vitro* cellular modelling is a useful tool and allows direct comparison between different JAK inhibitors, the *in vitro* results do not necessarily demonstrate the actual effects of JAK inhibitors *in vivo.* Indeed, our current results reveal novel information on the effects of tofacitinib on different JAK-STAT pathways *in vivo.*

Regarding the cytokine pathways that are preferentially suppressed by tofacitinib, both *in vitro* studies (17, 18, 26) and our current study show that tofacitinib potently suppresses JAK1/JAK3 -mediated signaling induced by the common-γ-chain cytokines IL-2, IL-4, IL-15 and IL-21. Our results also indicate that both IFN-α and IFN-γ responses were suppressed by tofacitinib to almost the same extent as responses induced by the common-γ-chain cytokines, while *in vitro* studies have demonstrated variable effects of tofacitinib on IFN signaling. Regarding that interferons are crucial mediators of antiviral responses, and that the risk of herpes zoster infection is significantly increased upon JAK inhibitor use (30), the significant inhibitory effect of tofacitinib on interferon-induced STAT1 signaling we revealed *in vivo* provides one possible mechanism for the herpes zoster infection susceptibility among tofacitinib users.

Our current *in vivo* results showed at least three inhibitory characteristics of tofacitinib that differed from the results obtained *in vitro*. First difference relates to the magnitude of inhibition of cytokine-induced STAT phosphorylation by tofacitinib. For example, the estimated daily average inhibition percentages of common-γ-chain cytokine (IL-2, IL-4, IL-15 and IL-21) responses in CD4^+^ T cells (18, 26) or lymphocytes (17) were comparable to the maximal inhibition percentages that we demonstrate in CD4^+^ T cells for these pathways *in vivo* (between 47 to 60%). Although the average and maximal inhibition percentages are not directly comparable, the current data nevertheless suggest that the *in vitro* studies may overestimate the inhibitory potential of tofacitinib on JAK-STAT pathway activation in RA patients *in vivo*. Our results also show that even after three months of tofacitinib treatment, cytokines can still induce STAT phosphorylation that exceeds the constitutive phosphorylation level. Therefore, the current results, although demonstrating a significant inhibitory effect of tofacitinib on constitutive and cytokine-induced STAT phosphorylation, further support the prevailing idea that oral dosing of JAK inhibitors, such as tofacitinib, allows only partial and reversible inhibition of JAK-STAT pathways (26, 31).

Second, the inhibitory effects of tofacitinib on IL-4, IL-6 and IL-10 -induced STAT phosphorylations in monocytes were lower *in vivo* than those described *in vitro* (17, 18, 26). IL-10 induced STAT3 phosphorylation demonstrated the lowest (10%) inhibition by tofacitinib in the current study. This indicates that the potent anti-inflammatory actions that IL-10 has on monocytes and macrophages (32) may not be efficiently suppressed by tofacitinib *in vivo*. In addition, regarding the potent anti-inflammatory and anti-arthritic effects of the IL-4/STAT6 pathway (33), the only modest *in vivo* potency of tofacitinib on IL-4-stimulated STAT6 phosphorylation, together with its weak effect on IL-10-stimulated STAT3 phosphorylation, may be advantageous features for the clinical efficacy of tofacitinib. It is also of note that in the present study, constitutive phosphorylation of STAT6, unlike that of the other STATs, was not decreased in any leukocyte subtype studied during 3-month tofacitinib treatment.

Third, *in vitro* data suggest that IL-6-induced STAT1 phosphorylation is more strongly inhibited by tofacitinib than IL-6-induced STAT3 phosphorylation in T cells (17, 18), whereas we demonstrate comparable inhibition of both IL-6-induced STAT pathways in CD4^+^ T cells of RA patients *in vivo*. Actually, regarding our previous finding that constitutive STAT3 phosphorylation is common in circulating CD4^+^ T cells in RA and associates with disease activity (8), one mechanism implementing the efficacy of tofacitinib in treating RA might be the relatively good inhibitory effect on CD4^+^ T cell STAT3 phosphorylation *in vivo*.

There are several possible explanations for the above-mentioned differences. We used cytokine concentrations that are likely to induce maximal STAT phosphorylation, whereas lower cytokine concentrations were generally used in *in vitro* studies. *In vivo* conditions also include more variables than *in vitro* studies; for example, in our study RA patients were also treated with csDMARDs, which may influence the magnitude of the inhibitory responses seen with tofacitinib, and furthermore, the conditions in which circulating leukocytes sense tofacitinib after oral administration are surely different from those in *in vitro* incubations. Finally, during three-month treatment, tofacitinib is likely to cause long-term effects that influence the inhibitory potential of tofacitinib and its selectivity towards different cytokine pathways and thus, results obtained by studying samples of healthy volunteers in *in vitro* studies may in principle be somewhat different from ours. In fact, we demonstrate that tofacitinib treatment suppresses the mRNA expression of certain components of the JAK-STAT pathway. In particular, the expression of inhibitory molecules *SOCS*s were downregulated, which may affect the overall inhibitory state of JAK-STAT pathways and that way also influence tofacitinib’s inhibitory potential *in vivo*.

The effect of tofacitinib on mRNA expression of *JAK*s, *STAT*s and *SOCS*s has not been extensively studied before. As cytokines are well-characterized inducers of *SOCS1-3* and *CIS1* expression (34), the observed decrease in their mRNA expression in the current study seems rational upon JAK inhibition. Decrease in *SOCS3* expression was the most prominent finding, and baseline *SOCS3* levels also correlated with the treatment response. This is an interesting observation regarding that STAT3-regulated *SOCS3* expression in CD4^+^ T cells has been shown to be elevated in a cohort of 161 treatment-naïve early arthritis patients (35) and in PBMCs from patients with active RA (36). Our data suggests that tofacitinib treatment also slightly but significantly decreases *STAT3*, *STAT4*, *STAT5A*, *JAK1* and *JAK3* expression. However, as STAT3 protein levels were not repressed after the 3-month tofacitinib treatment, the mechanistic significance of the changes in STAT mRNA expression remains elusive. As tofacitinib has been shown to decrease expression of extracellular proteases, such as matrix metalloprotease 1 in rheumatoid arthritis synovial fibroblasts (37), it is possible that the inhibitory effect of tofacitinib extends also to intracellular protein degradation, such as the ubiquitin-proteasome system. This could explain the unchanged STAT3 protein level.

In order to estimate how tofacitinib-specific the observed decrease in constitutive and cytokine-induced phosphorylation of STATs is, we compared the current results to our previous results on csDMARD-unresponsive RA patients treated with biologic DMARD (11). During biological DMARD treatment, IL-4 -induced pSTAT6 was downregulated. Interestingly, the effect was clearly weaker than that shown with tofacitinib in the current study. Strikingly, neither constitutive nor IFN-γ-stimulated pSTAT1 showed significant changes during biological DMARD treatment, while the correspondent pSTAT1 levels fell significantly by tofacitinib use. For example, tofacitinib downregulated IFN-γ-stimulated pSTAT1 in monocytes by 34% and in CD4^+^ T cells by 49%. Although results from different studies are not directly comparable, the data so far suggest that decrease in STAT phosphorylation is not a general effect achieved by any RA medication.

The association between baseline STAT phosphorylations and treatment response to tofacitinib was also examined. Baseline constitutive pSTAT1 and pSTAT3 levels in monocytes and CD4^+^ T cells, and pSTAT5 levels in CD4^+^ T cells correlated positively with the response. The strongest correlation was seen with pSTAT3. The magnitude of decrease in pSTAT3 levels following tofacitinib treatment also correlated positively with treatment response. As we and others have previously shown that constitutive pSTAT1, pSTAT3 and pSTAT5 levels in T cells and pSTAT3 levels in monocytes are elevated in RA patients (7, 9, 10), tofacitinib obviously targets several RA-associated leukocyte signaling aberrations successfully. The comparison of STAT phosphorylation levels between controls and RA patients (historical data) to those between tofacitinib-treated and untreated RA patients derived from the current study suggests that the increased constitutive phosphorylation of STATs in patients with RA is reversed to a significant degree *in vivo* by tofacitinib. However, as healthy controls were not included in the current study, it remains unresolved whether the patients’ STAT phosphorylation levels reached those of healthy individuals.

The only inverse correlation we found between baseline pSTAT levels and treatment response concerned STAT4. In this context it may be noteworthy that STAT4 is able to cause sustained expression of genes that increase sensitivity to IL-18 (38). IL-18 signaling does not take place *via* JAK-STAT pathways and hence, is not directly affected by tofacitinib. Both IL-18 receptor and STAT4 deficiency have suppressed the severity of arthritis in a murine model of RA (39, 40). Furthermore, STAT4 represses the genes of the Th2 cytokines IL-5 and IL-13 (41), both of which have been associated with arthritis-limiting capacity and decreased progression to fully established RA (33). Hence, STAT4 phosphorylation measured in the patients’ leukocytes at baseline may be associated with immunological features that are not so easily amended by tofacitinib. It is also notable that genetic variation of STAT4 has been associated with the risk of autoimmune diseases like systemic lupus erythematosus and rheumatoid arthritis (42). It remains to be elucidated whether STAT4 variants are associated with STAT4 phosphorylation levels and RA patients’ response to tofacitinib.

The effect of tofacitinib treatment on the pathobiology of rheumatoid synovium has been studied by Boyle et al. using immunohistochemistry (43). Their results showed a positive correlation between 4-month clinical improvement and reductions in STAT1 and STAT3 phosphorylation at day 28 (43). However, unlike ours, their results did not show associations between baseline STAT phosphorylation levels and the clinical response. This discrepancy might be due to methodological differences between the two studies, but it is also possible that constitutive and cytokine-stimulated pSTAT levels in circulating leukocytes, rather than in synovial cells, represent a more sensitive sensor of the overall immunological state. Nevertheless, further studies are needed to show whether STAT phosphorylation or *SOCS3* expression could be used as a biomarker to predict clinical response to tofacitinib treatment.

The strengths of the current study are: 1) prospective study on well-characterized RA patients in whom multiple JAK-STAT signaling pathways were analyzed close to *in vivo* conditions before and three months after starting tofacitinib therapy; 2) the use of whole blood flow cytometric assay, which minimizes inappropriate cell signaling pathway activation due to sample handling. The limitation of the study is that the relatively small patient cohort enables only predictive conclusions on the clinical significance of the observed association between baseline immunological parameters and treatment response. Even though it needs to be confirmed in further studies if the baseline JAK-STAT signaling profile has prognostic value for tofacitinib treatment responses, this study significantly adds to our understanding about the mechanisms of tofacitinib function in RA patients receiving this treatment.

## Data Availability Statement

The original contributions presented in the study are included in the article/**Supplementary Material**. Further inquiries can be directed to the corresponding author.

## Ethics Statement

The studies involving human participants were reviewed and approved by National Committee on Medical Research Ethics (TUKIJA) and Finnish Medicines Agency Fimea. The patients/participants provided their written informed consent to participate in this study.

## Author Contributions

MP, AV, KK, AKuu, ML-R, OS, and PI planned the study. RP, AKin, and PI recruited the patients. MP, KK, and AKur collected the data. MP, AV, AKuu, and AKur analyzed the results. MP, KK, AV, AKuu, and PI wrote the manuscript and all authors gave valuable comments on the manuscript. All authors contributed to the article and approved the submitted version.

## Funding

This study was supported by a grant from Pfizer. The funder was not involved in the study design, collection, analysis, interpretation of data, the writing of this article or the decision to submit it for publication.

## Conflict of Interest

MP has received a personal fee from Pfizer and is currently a part-time employee of MedEngine. KK has received a grant from Pfizer. RP has received personal fees from Pfizer, Eli Lilly and Company and Janssen. AKin has received non-financial support from Sandoz, Mylan, Celgene and Bristol-Myers Squibb. PI has received a grant from Pfizer and personal fees from Pfizer, Eli Lilly and Company, Abbvie and Roche and non-financial support from Abbvie and Roche. OS has received personal fees from Pfizer and Abbvie and holds patents on JAK kinases, US Patent no. 5,728,536, US patent no. 8,841,078, AU 2011214254, CAN 2789186, and EPO 11741946.5. OS is employed by Fimlab Laboratories Ltd.

The remaining authors declare that the research was conducted in the absence of any commercial or financial relationships that could be construed as a potential conflict of interest.

## Publisher’s Note

All claims expressed in this article are solely those of the authors and do not necessarily represent those of their affiliated organizations, or those of the publisher, the editors and the reviewers. Any product that may be evaluated in this article, or claim that may be made by its manufacturer, is not guaranteed or endorsed by the publisher.
