# Supplementary material for: Tofacitinib Suppresses Several JAK-STAT Pathways in Rheumatoid Arthritis In Vivo and Baseline Signaling Profile Associates With Treatment Response
Source: Front Immunol. 2021 Sep 24;12:738481. doi: 10.3389/fimmu.2021.738481 (PMC8498592; doi:10.3389/fimmu.2021.738481)
Supplement: Supplementary file 1 [file DataSheet_1.docx]

Supplementary Material

**Supplementary Table 1**. Recombinant cytokines and their manufacturers.

| **Cytokine** | **Company** |
| --- | --- |
| IL-2 | Peprotech, Rocky Hill, NJ, USA |
| IL-4 | Peprotech |
| IL-6 | Peprotech |
| IL-7 | Peprotech |
| IL-10 | R&D Systems, Minneapolis, MN, USA |
| IL-15 | Peprotech |
| IL-21 | Peprotech |
| IFN-α | Cell Signaling Technology, Danvers, MA, USA |
| IFN-γ | Peprotech |

**Supplementary Table 2**. Flow cytometry antibodies, conjugates, clones, and manufacturers.

| **Antibody** | **Conjugate** | **Clone** | **Company** |
| --- | --- | --- | --- |
| CD33 | APC | P67.7 | Becton, Dickinson and Company (BD), Franklin Lakes, NJ, USA |
| CD3 | FITC | SK7 | BD |
| CD4 | APC e-Fluor | SK3 | eBioscience, Santa Clara, CA, USA |
| CD20 | PE-Cy7 | H1(FB1) | BD |
| STAT1 | PE | 1/Stat1 | BD |
| STAT3 | PE | M59-50 | BD |
| pSTAT1 | PE | 4a | BD |
| pSTAT3 | PE | 4/P-STAT3 | BD |
| pSTAT4 | PE | 38/p-Stat4 | BD |
| pSTAT5 | PE | 47 | BD |
| pSTAT6 | PE | 18/P-Stat6 | BD |

**Supplementary Table 3**. Primer sequences.

| **Gene** | **Forward primer sequence (5’–3’)** | **Reverse primer sequence (5’–3’)** |
| --- | --- | --- |
| STAT1 | TCACATTCACATGGGTGGAG | CAAAGGCATGGTCTTTGTCA |
| STAT3 | TCACATGCCACTTTGGTGTT | GCAATCTCCATTGGCTTCTC |
| STAT4 | GGCAATTGGAGAAACTAGAGG | AGGGTGGGTTGGCATACAT |
| STAT5A | GCCAGATGCAGGTGCTGTA | GGGATTGTCCAAGTCAATGG |
| STAT5B | GCGTTATATGGCCAGCATTT | CTGGTGCTCTGCCTTCTTCT |
| STAT6 | GGAAGGGCACTGAGTCTGTC | GGCTTTGGCATTGTTGTCTT |
| JAK1 | CATGGTGGAAGAGTTTGTGGA | CAGCTGTTTGGCAACTTTGAATT |
| JAK2 | CCGCCGGGTTTCAGAAG | GAAGAGGTGGATGTTCCCTCC |
| JAK3 | AGTGGGACTTTCCTCTCGC | CTCTTCACTTGGAGGTGCCAT |
| TYK2 | CCCATGGCTTGGAAGATGGT | ACTCAGCTTGATGAAGGGGC |
| SOCS1 | CTGGGATGCCGTGTTATTTT | TAGGAGGTGCGAGTTCAGGT |
| SOCS2 | CAGGGAATGGCAGAGACACT | TGGCAGAGAGAGAAGGGATG |
| SOCS3 | GCCACCTACTGAACCCTCCT | ACGGTCTTCCGACAGAGATG |
| CIS1 | AGCCCAGACAGAGAGTGAGC | TGACAGCGTGAACAGGTAGC |
| β-actin | TGGGACGACATGGAGAAAAT | AGAGGCGTACAGGGATAGCA |

**Supplementary Table 4.** csDMARD treatment of the patients at baseline.

| **Patient** | | **Methotrexate** | **Sulphasalazine** | **Hydroxychloroquine** | **Other csDMARD** | **Prednisolone** |
| --- | --- | --- | --- | --- | --- | --- |
| 1 | 15 mg weekly po | | 2000 mg daily | 300 mg daily |  |  |
| 2 | 20 mg weekly po | |  |  |  | 10 mg daily |
| 3 | 25 mg weekly po | | 2000 mg daily |  |  |  |
| 4 | 15 mg weekly sc | | 2000 mg daily |  |  |  |
| 5 |  | | 2000 mg daily | 300 mg daily |  |  |
| 6 | 15 mg weekly po | | 2000 mg daily | 300 mg daily |  | 2.5 mg daily |
| 7 | 25 mg weekly sc | |  | 300 mg 6 days/week |  |  |
| 8 | 20 mg weekly sc | |  | 300 mg 6 days/week |  | 2.5 mg daily |
| 9 | 25 mg weekly sc | | 2000 mg daily | 300 mg 4-5 days/week |  |  |
| 10 | 25 mg weekly sc | | 1500 mg daily | 300 mg 6 days/week |  | 2.5 mg daily |
| 11 |  | |  | 300 mg daily |  |  |
| 12 | 20 mg weekly sc | |  |  |  | 5 mg daily |
| 13 |  | | 2000 mg daily |  | leflunomide 20 mg daily | 5 mg daily |
| 14 |  | |  | 300 mg daily | azathioprine 50 mg daily |  |
| 15 | 25 mg weekly po | | 2000 mg daily | 300 mg daily |  | 5 mg daily |
| 16 | 25 mg weekly sc | | 2000 mg daily | 300 mg daily |  | 7.5 mg daily |

csDMARD, conventional synthetic disease-modifying antirheumatic drug; po, oral administration; sc, subcutaneous injection

**Supplementary Table 5**. The difference in constitutive STAT1, STAT3 and STAT5 phosphorylation in circulating monocytes and CD4-positive (CD4^+^) and -negative (CD4^-^) T lymphocytes between RA patients and healthy controls (left panels), and between RA patients before and after tofacitinib treatment (right panels). Comparison between healthy controls (n=11-13) and rheumatoid arthritis patients (n=12-14) was performed on data derived from previous studies (references 7 and 10), and comparison between untreated and tofacitinib-treated RA patients (n=16) on data from the current study. The experimental methods used in the previous and current studies were similar, but for better comparison of two separate studies, mean fluorescence intensity ratios (MFIR) were calculated by dividing the median fluorescence intensity (MFI) value of each pSTAT with MFI value of mouse IgG isotype control. Both the absolute differences (**Δ**MFIR) and percentage differences in MFIR between the groups are presented. pSTAT levels of RA patients and healthy controls were compared using Mann-Whitney test, and pSTAT levels before and after tofacitinib treatment with Wilcoxon test and p-values are shown in the Table.

|  |  | **RA patients vs healthy controls** | | | **RA patients before vs after tofacitinib treatment** | | |
| --- | --- | --- | --- | --- | --- | --- | --- |
| **pSTAT** | **Cell type** | **ΔMFIR**  **(RA patients – healthy controls)** | **Percentage difference in MFIR** | **p** | **ΔMFIR (before tofacitinib – after tofacitinib)** | **Percentage difference in MFIR** | **p** |
| pSTAT1 | Monocytes | 0.00 | 0 | 0.846 | 0.68 | 26 | 0.002 |
|  | CD4^+^ T cells | 0.52 | 20 | 0.020 | 1.87 | 34 | 0.002 |
|  | CD4^-^ T cells | 0.22 | 11 | 0.139 | 1.00 | 30 | 0.002 |
| pSTAT3 | Monocytes | 1.38 | 39 | 0.001 | 4.41 | 34 | 0.005 |
|  | CD4^+^ T cells | 2.43 | 55 | <0.001 | 12.21 | 44 | 0.003 |
|  | CD4^-^ T cells | 0.71 | 31 | 0.024 | 0.95 | 23 | 0.019 |
| pSTAT5 | Monocytes | 0.03 | 2 | 0.538 | 0.49 | 4 | 0.518 |
|  | CD4^+^ T cells | 1.54 | 38 | <0.001 | 9.43 | 41 | 0.003 |
|  | CD4^-^ T cells | 0.49 | 19 | 0.006 | 1.31 | 10 | 0.187 |

*data derived from references 7 and 10. MFIR, mean fluorescence intensity ratio; (p)STAT, (phosphorylated) signal transducer and activator of transcription.

**Supplementary Figure 1.** Correlation of DAS28 improvement during tofacitinib and csDMARDs treatment and basal mean fluorescence intensity (MFI) levels at baseline of A) monocyte and B) CD4^+^ T cell pSTAT1. C) monocyte and D) CD4^+^ T cell pSTAT3. E) monocyte and F) CD4^-^ T cell pSTAT4. and G) CD4^+^ T cell pSTAT5. Lines of best fit are shown. csDMARD, conventional systemic disease-modifying antirheumatic drug; DAS28, composite Disease Activity Score for 28 joints based on the C-reactive protein level (DAS28-4[CRP]); (p)STAT, (phosphorylated) signal transducer and activator of transcription.

**Supplementary Table 6.** Correlation of baseline demographic. clinical and laboratory variables with DAS28 improvement during 3-month treatment with tofacitinib and csDMARDs. Spearman correlation coefficients (r) are used. Results with p-values ≤0.05 are shown in bold.

|  | **r** | **p** |
| --- | --- | --- |
| Swollen joint count. 0-46 | 0.167 | 0.537 |
| Tender joint count. 0-46 | 0.124 | 0.647 |
| Swollen joint count. 0-28 | -0.051 | 0.852 |
| Tender joint count. 0-28 | 0.297 | 0.264 |
| General health. VAS. 0-100 mm | 0.069 | 0.799 |
| Pain. VAS. 0-100 mm | 0.130 | 0.632 |
| Physician’s assessment. VAS. 0-100 mm | -0.155 | 0.566 |
| HAQ disability index. 0-3 | -0.235 | 0.381 |
| DAS28 | 0.549 | **0.028** |
| Plasma C-reactive protein. mg/l | 0.554 | **0.026** |
| Blood haemoglobin. g/l | -0.105 | 0.699 |
| Blood leukocyte count. ×10^9^/l | -0.043 | 0.875 |
| Blood neutrophil count. ×10^9^/l | 0.103 | 0.704 |
| Blood lymphocyte count. ×10^9^/l | -0.397 | 0.128 |
| Blood platelet count. ×10^9^/l | 0.227 | 0.399 |
| Plasma alanine aminotransferase. U/l | -0.362 | 0.168 |
| Plasma creatinine. µmol/l | 0.121 | 0.656 |

DAS28, composite Disease Activity Score for 28 joints based on the C-reactive protein level (DAS28-4[CRP]); HAQ, Health Assessment Questionnaire; IQR, interquartile range; VAS, visual analogue scale

**Supplementary Table 7.** Correlation of change in pSTAT and total STAT1 and STAT3 levels with DAS28 improvement during 3-month treatment with tofacitinib and csDMARDs. Spearman correlation coefficients (r) are used. Results with p-values ≤0.05 are shown in bold. Spaces left empty denote the cases in which the stimulated phosphorylation level does not differ from the basal level

|  |  |  | **Cell type** | | | | | | | | | | |
| --- | --- | --- | --- | --- | --- | --- | --- | --- | --- | --- | --- | --- | --- |
|  |  |  | **Monocytes** | |  | **CD4^+^ T cells** | |  | **CD4^-^ T cells** | |  | **B cells** | |
| **Molecule** | **Stim.** |  | **r** | **p** |  | **r** | **p** |  | **r** | **p** |  | **r** | **p** |
| pSTAT1 | None |  | 0.313 | 0.256 |  | 0.407 | 0.132 |  | 0.375 | 0.168 |  | 0.000 | 1.000 |
| pSTAT1 | IFN-γ |  | -0.009 | 0.974 |  | 0.450 | 0.080 |  |  |  |  | 0.079 | 0.770 |
| pSTAT1 | IFN-α |  | -0.229 | 0.393 |  | -0.285 | 0.284 |  | -0.303 | 0.254 |  | -0.256 | 0.339 |
| pSTAT1 | IL-6 |  | -0.103 | 0.704 |  | 0.044 | 0.871 |  |  |  |  |  |  |
| pSTAT3 | None |  | **0.600** | **0.014** |  | **0.682** | **0.004** |  | 0.009 | 0.974 |  | 0.041 | 0.880 |
| pSTAT3 | IL-6 |  | 0.212 | 0.431 |  | 0.297 | 0.264 |  | 0.056 | 0.837 |  | 0.124 | 0.649 |
| pSTAT3 | IL-10 |  | -0.050 | 0.854 |  | 0.074 | 0.787 |  | 0.112 | 0.680 |  | 0.482 | 0.058 |
| pSTAT3 | IL-21 |  |  |  |  | 0.174 | 0.520 |  | 0.168 | 0.535 |  | -0.256 | 0.339 |
| pSTAT4 | None |  | -0.162 | 0.549 |  | -0.135 | 0.617 |  | -0.056 | 0.837 |  | -0.132 | 0.625 |
| pSTAT4 | IFN-α |  |  |  |  | -0.368 | 0.161 |  | -0.385 | 0.141 |  |  |  |
| pSTAT5 | None |  | -0.374 | 0.154 |  | 0.071 | 0.795 |  | -0.282 | 0.289 |  | -0.200 | 0.458 |
| pSTAT5 | IL-2 |  |  |  |  | 0.109 | 0.688 |  | 0.021 | 0.940 |  |  |  |
| pSTAT5 | IL-7 |  |  |  |  | -0.026 | 0.922 |  | -0.341 | 0.196 |  |  |  |
| pSTAT5 | IL-15 |  |  |  |  | 0.021 | 0.940 |  | -0.121 | 0.656 |  |  |  |
| pSTAT6 | None |  | 0.106 | 0.696 |  | 0.110 | 0.684 |  | -0.077 | 0.778 |  | 0.232 | 0.387 |
| pSTAT6 | IL-4 |  | -0.024 | 0.931 |  | 0.053 | 0.846 |  | -0.226 | 0.399 |  | -0.253 | 0.345 |
| STAT1 | None |  | -0.368 | 0.161 |  | -0.224 | 0.405 |  | -0.168 | 0.535 |  | -0.124 | 0.649 |
| STAT3 | None |  | -0.164 | 0.558 |  | -0.221 | 0.428 |  | -0.268 | 0.334 |  | -0.239 | 0.390 |

csDMARD, conventional systemic disease-modifying antirheumatic drug; DAS28, composite Disease Activity Score for 28 joints based on the C-reactive protein level (DAS28-4[CRP]); IFN, interferon; IL, interleukin; (p)STAT, (phosphorylated) signal transducer and activator of transcription; stim., stimulation

**Supplementary Table 8.** Correlation of mRNA expression levels of JAK-STAT pathway genes in PBMCs with disease activity. A. expression at baseline vs the change from baseline in DAS28 during 3-month treatment with tofacitinib and csDMARDs; B. change in expression vs the change from baseline in DAS28 during 3-month treatment with tofacitinib and csDMARDs. Spearman correlation coefficients (r) are used. Results with p values ≤0.05 are shown in bold.

|  |  |  | **A** | |  | **B** | |
| --- | --- | --- | --- | --- | --- | --- | --- |
| **Transcript** |  |  | **r** | **p** |  | **r** | **p** |
| *STAT1* |  |  | -0.065 | 0.812 |  | 0.094 | 0.729 |
| *STAT3* |  |  | -0.021 | 0.940 |  | -0.047 | 0.863 |
| *STAT4* |  |  | -0.056 | 0.837 |  | 0.188 | 0.485 |
| *STAT5A* |  |  | -0.297 | 0.264 |  | 0.309 | 0.244 |
| *STAT5B* |  |  | -0.135 | 0.617 |  | -0.147 | 0.587 |
| *STAT6* |  |  | -0.015 | 0.957 |  | -0.224 | 0.405 |
| *JAK1* |  |  | -0.388 | 0.137 |  | 0.376 | 0.151 |
| *JAK2* |  |  | -0.212 | 0.431 |  | 0.103 | 0.704 |
| *JAK3* |  |  | 0.229 | 0.393 |  | -0.194 | 0.471 |
| *TYK2* |  |  | 0.153 | 0.572 |  | -0.121 | 0.656 |
| *SOCS1* |  |  | -0.044 | 0.871 |  | 0.462 | 0.072 |
| *SOCS2* |  |  | -0.132 | 0.625 |  | 0.450 | 0.080 |
| *SOCS3* |  |  | **0.532** | **0.034** |  | -0.229 | 0.393 |
| *CIS1* |  |  | -0.174 | 0.520 |  | 0.471 | 0.066 |

CIS1, cytokine-inducible SH2 domain-containing protein; csDMARD, conventional systemic disease-modifying antirheumatic drug; DAS28, composite Disease Activity Score for 28 joints based on the C-reactive protein level (DAS28-4[CRP]); JAK, Janus kinase; PBMC, peripheral blood mononuclear cell; SOCS, suppressor of cytokine signaling; STAT, signal transducer and activator of transcription; TYK2, tyrosine kinase 2
